# LncRNA SNHG4 Modulates EMT Signal and Antitumor Effects in Endometrial Cancer through Transcription Factor SP-1

**DOI:** 10.3390/biomedicines11041018

**Published:** 2023-03-27

**Authors:** Lee Kyung Kim, Sun-Ae Park, Eun Ji Nam, Young Tae Kim, Tae-Hwe Heo, Hee Jung Kim

**Affiliations:** 1 Laboratory of Pharmacoimmunology, Integrated Research Institute of Pharmaceutical Sciences and BK21 FOUR Team for Advanced Program for SmartPharma Leaders, College of Pharmacy, The Catholic University of Korea, 43 Jibong-ro, Bucheon-si 14662, Republic of Korea; 2 Institute of Women’s Life Medical Science, Division of Gynecologic Oncology, Department of Obstetrics and Gynecology, Yonsei University College of Medicine, Seoul 03722, Republic of Korea

**Keywords:** SNHG4, EMT signal, SP-1, transcription factor, endometrial cancer

## Abstract

Long non-coding RNAs (lncRNAs) are implicated in the initiation and progression of a variety of tumors, including endometrial cancer. However, the mechanisms of lncRNA in endometrial cancer formation and progression remain largely unknown. In this study, we confirmed that the lncRNA SNHG4 is upregulated in endometrial cancer and correlates with lower survival rates in endometrial cancer patients. Knock-down of SNHG4 significantly reduced cell proliferation, colonization, migration, and invasion in vitro, as well as modulating the cell cycle and reduced tumor growth of endometrial cancer in vivo. In addition, the effect of SNHG4 by the transcription factor SP-1 was confirmed in vitro. We found in this study that SNHG4/SP-1 plays an important role in endometrial cancer progression and may be used as a potential therapeutic and prognostic biomarker for endometrial cancer.

## 1. Introduction

Endometrial cancer is a common gynecological cancer, with over 319,000 cases diagnosed worldwide and over 76,000 deaths per year [1]. Most endometrial cancers diagnosed at an early stage have favorable outcomes. However, when endometrial cancer metastasizes and recurs, the clinical outcome deteriorates significantly. Endometrial cancer diagnosis is confirmed by biopsy or dilation and curettage, and staging and histology are confirmed by surgery. Patients with endometrial cancer can undergo surgery alone or undergo vaginal brachytherapy (BRT) or external beam radiation therapy (EBRT), with the addition of platinum-based therapy for high-risk stage I and stage II patients [2]. Endometrial cancer has a favorable prognosis with a 5-year overall survival (OS) of 74–95%, but patients with advanced or metastatic endometrial cancer still have a poor prognosis with a 5-year OS of 15–17% due to tumor metastasis and recurrence [3]. Therefore, there is a need for an effective strategy for the diagnosis and treatment of endometrial cancer [4,5].

ncRNA is mainly composed of ribosomal RNA (rRNA), long noncoding RNA (lncRNA), transfer RNA (tRNA), microRNA (miRNA), small nuclear RNA (snRNA), circRNA (circRNA), small nucleolar RNA (snoRNA), and piwi-interacting RNA (piRNA) [6]. Long non-coding RNAs (lncRNAs) are non-protein-coding transcripts greater than 200 nucleotides and are aberrantly expressed in a wide variety of cancers and play important roles in regulating gene expression [7]. LncRNAs can act as guides to promote or repress transcription and serve as scaffolds by interacting with chromatin modification complexes. In human tumors, lncRNAs are modulators of several cancer phenotypes, including tumor cell proliferation, motility, invasion, and metastasis [8,9]. Therefore, it is believed that lncRNAs have a potential impact in tumor development and that lncRNAs can be used as novel cancer biomarkers and serve as possible therapeutic targets for this neoplasia.

Small nucleolar RNA host gene 4 (SNHG4), a lncRNA, has been found to be aberrantly expressed in several tumors. LncRNA SNHG4, which contains exons and introns, is the host gene for snoRNA (small nucleolar RNAs). The introns are mostly processed by snoRNA, and exons are recombined to play a role in the cytoplasm. The SNHG family has been reported to have 22 members, from SNHG1 to SNHG22. They play an important role in cancer and other diseases in humans [10,11,12]. SNHG4 is known to play an oncogenic role in tumors, and decreased expression of SNHG4 may inhibit tumor cell proliferation; therefore, it is a potential target for cancer treatment. Although research on SNHG4 in human diseases is increasing, there are no research reports on endometrial cancer as of yet. In addition, a gap still exists between research and clinical research relationships [13].

Transcription factors (TFs) are known to play a pivotal role in regulating cell donation, including inhibition and alteration of gene expression and cell activation. TFs can lead to cellular induction of tumorigenesis; mutations in cancer result from changes in the various protein functions and transcription factors that regulate proteins, resulting in phenotypic changes [14]. One of the transcription factors, SP-1, is a member of the SP family of proteins that contain the C2H2 zinc finger domain and is thought to be involved in the transcriptional regulation of housekeeping genes. However, evidence suggests a specific role for SP1 in regulating genes’ hallmarks of cancer and genes required for development and differentiation [15]. SP-1 is a gene downstream of the lncRNA/miR/mRNA axis and has been found to be regulated by lncRNA-miR crosstalk in various cancers, including lung adenoma and non-small cell lung cancer [16,17]. It is known that the activity of SP-1 regulates lncRNA in breast and colorectal cancer and promotes chemical resistance and cancer progression [18,19].

In this study, we tried to confirm the expression of lncRNA SNHG4 as a predictive marker for endometrial cancer and to study its relationship with transcription factor SP-1.

## 2. Materials and Methods

### 2.1. Cell Lines and Cell Culture

We purchased the endometrial cancer cell lines Ishikawa and ECC1 (Essai de Corrosion Cyclique1) cell lines from Sigma-Aldrich (99040201, Dorset, UK) and ATCC (CRL-2923, Rockville, MD, USA). For cell growth conditions, MEM or RPMI 1640 medium was used, to which 10% *v*/*v* fetal bovine serum (FBS, Corning, Oneonta, NY, USA) and 1% *v*/*v* penicillin–streptomycin (PS, Gibco, Waltham, MA, USA) mixture (source) were added. MCF10A human epithelial cells (the mammary gland with fibrocystic breasts as a control) were obtained from Prof. Kwak MK (The Catholic University of Korea). The cell lines were maintained at 37 °C in humidified air with 95% air and 5% CO_2_, and no cells exceeded 20 passages. ECC1 cells were cultured in RPMI 1640 (Welgene, Daegu, Republic of Korea) and Ishikawa cells in MEM (Welgene, Daegu, Republic of Korea).

### 2.2. Serum Sample

Patient samples were performed with the approval of the review board forhuman research of Yonsei University Hospital. Samples were obtained from 100 patients with endometrial cancer who underwent surgery from September 2012 to December 2019 and a control group of 26 patients with benign gynecological disease. Progression-free survival (PFS) was defined as the interval between surgical and progression data confirmed by imaging studies. The present study was approved by the Ethics Committee of Yonsei Severance Hospital (ethic code: 4-2012-0363), and informed consent was obtained from all patients. 

### 2.3. Transfection of SNHG4

siRNAs (SNHG4 and SP-1) were purchased from Genolution (Genolution Phar-maceutical Inc., Seoul, Republic of Korea). Cells were added to a 6-well plate at a density of 5 × 10^5^ cells/well. To transfect cells, a G-Fectin Kit (Genolution Pharmaceutical Inc.) was used according to the manufacturer’s instructions with 30 nM siRNA in phosphate buffered saline (PBS). After 48 h transfected cells were used for in vitro assays. siRNA sequences used are shown in Supplementary Table S1.

### 2.4. Trans-Well Migration Assay

Migration assays were designed using a 6.5 mm-diameter trans-well plate (Corning Costar, Cambridge, MA, USA) with an 8 μm pore filter. Cells (5 × 10^5^) were seeded into the upper chamber of serum-free medium, and complete medium was added to the lower chamber. Cells transfected with SNHG4 and SP-1 were incubated for 48 h in a transfer chamber set at 37 °C and 5% CO_2_. A cotton swab was used to remove non-invasive cells from the upper chamber. Cells invading the lower surface of the filter were stained (Diff Quik, Sysmes, Kobe, Japan), and stained cells were counted using ImageJ software. This analysis was repeated at least three times.

### 2.5. Matrigel Invasion Assay

For the Matrigel invasion chamber (pore size: 8 mm, BD Bioscience, Lowell, MA, USA) was according to the manufacture’s protocol. Cells were seeded in a 6-well plate at a density of 1 × 10^6^, they were treated with 30 nM of SNHG4 and SP-1 transfected cells for 48 h. Next, 5 × 10^5^ cells were seeded in the upper chamber with serum-free medium and complete medium (10% FBS, 1% penicillin-streptomycin) was added to the lower chamber. The chamber was incubated for 48 h in an incubator set at 37 °C and 5% CO_2_. After 48 h, non-invasive cells on the top of the chamber were removed with a cotton swab. The number of cells invading under the filter was determined using a staining reagent (Diff Quik, Kobe, Japan) and the number of stained cells was counted using ImageJ software

### 2.6. Colony Formation

ECC1 and Ishikawa cells were transfected with 30 nM of SNHG4 and SP-1 for 48 h and then seeded at 1 × 10^5^ cells per well in a 6-well plate to proceed with colony formation. After culturing for 2 weeks, cells were fixed with cold methanol (90%) for 5 min and stained with crystal violet (0.5% crystal violet in phosphate buffered saline (PBS), Samcheon, Gyeonggi-do, Republic of Korea) for 30 min. Colony formation was observed under a microscope after staining and results were quantified using ImageJ software.

### 2.7. Cell Proliferation Assay

Cell proliferation ability and cytotoxicity were evaluated using the Cell Counting Kit-8 (CCK-8). After seeding 1 × 10^5^ cells/well in 48 well plates, cells were transfected with 30 nM of SNHG4 and SP-1. For CCK-8 analysis, 20 μL of CCK-8 (CCK-8, Dojindo, Kumamoto, Japan) was added for each time period (0, 24, 48, 72 h), reacted for 1 h, and the optical density (OD) value was measured.

### 2.8. Cell Cycle Assay

For cell cycle analysis, after ECC-1 and Ishikawa cells were transfected with SNHG4 for 48 h, they were quantified by flow cytometry. Experiments were conducted using PI (BD Pharmingen, San Diego, CA, USA) according to the manufacturer’s instructions. Cell cycles were then analyzed using flow cytometry. After flow cytometry, they were analyzed with BD FACSDiva software version 6.2. Cell cycle cells were calculated after FACS analysis. 

### 2.9. Real-Time PCR

The total RNA of serum was extracted with an RNA extraction kit (QIAGEN, Hilden, Germany). Approximately 5 mL of the serum was obtained per patient, and approximately 0.5 μg of RNA was extracted from the separated serum. 

For cDNA synthesis, total RNA was synthesized into cDNA using a reverse transcription reagent kit (Bioline, London, UK) according to the manufacturer’s instructions. Real-time PCR analysis was performed using the SYBR Green Real-Time PCR Kit (Bioline, London, UK). Conditions for amplification were as follows: initial denaturation at 95 °C for 3 min, 40 cycles of denaturation at 95 °C for 15 s, annealing at 60 °C for 60 s, and elongation at 72 °C for 60 s, 72 °C Final elongation for 5 min. qRT-PCR was performed using the CFX96 Touch Real-Time PCR Detection System (Biorad, Hercules, CA, USA).

Normalization was expressed as U6, 18 sr and Gapdh, and relative mRNA expression changes were calculated by the 2-ΔΔCT method. All experiments were performed in triplicate and the PCR target primer sequences are shown in Supplementary Table S2.

### 2.10. Transcription Factor (TF) Binding Motif

We identified transcription factors using the Alibaba 2.1 database (http://gene-regulation.com/pub/programs/alibaba2/ (accessed on 9 May 2022)) to identify transcription factor (TF) binding motifs in SNHG4. Using the JASPAR database (https://jaspar.genereg.net/ (accessed on 9 May 2022)), we checked which part of the transcription factor binds.

### 2.11. Western Blotting Analysis

Cells were lysed, and protein was extracted with RIPA buffer (Biosesang, Seongnam, Republic of Korea). The protein concentration was measured using the Pierce BCA Protein Assay Kit (Thermo Fisher Scientific Inc, Cramlington, UK). Protein samples were boiled with 5× sample buffer, further resolved in 10% SDS/polyacrylamide gel, and then electrophoretically transferred to polyvinylidene difluoride (PVDF) membranes (Atto, Tokyo, Japan). After being blocked with 5% skim milk in 1× Tris-buffered saline containing 0.1% Tween 20 (pH 7.6) for 1 h 30 m at room temperature, the membrane was stirred continuously and incubated with the following primary antibodies: N-cadherin (1:1000, 4061 s, Cell Signaling, Beverly, MA, USA), E-cadherin (1:1000, 3192 s, Cell Signaling), β-catenin (1:1000, 9562 s, Cell Signaling), Vimentin (1:1000, 3932 s, Cell Signaling), Snail (1:1000, 3879 s, Cell Signaling), NOTCH1 (1:1000, 3608 s, Cell Signaling), SP-1 (1:1000, 9389 s, Cell Signaling), and β-actin (1:1000, 8457 s, Cell Signaling) overnight at 4 °C. Next, the membranes were further incubated with secondary antibody Goat anti-Rabbit IgG (H + L) Secondary Antibody, HRP (1:3000, 7074 s, Cell Signaling) to detect the immunoreactivity of the proteins. The Western blotting membranes were scanned using a Bio-Rad ChemiDoc image system to visualize protein bands.

### 2.12. Xenograft

The experiment was conducted with BALB/c nude mice (*n* = 4; 5 weeks old; Orient Bio, Seongnam, Republic of Korea) at a constant temperature and humidity according to the Catholic University protocol. Experimental animal procedures were approved by the Institutional Care and Use Committee of The Catholic University of Korea (approval number: CUK-IACUC-2019-026, permit code, 29 May 2019) and complied with legal obligations and federal guidelines for care and maintenance. ECC1 cells (1 × 10^6^) were subcutaneously injected into the dorsal scapular region of BALB/c nude mice. Tumor size was measured every 2 days using calipers and tumor volume was measured using a simplified equation to estimate the spheroid (length × width × 0.5). End of experiment was harvested 11 days after cell injection.

### 2.13. Hematoxylin and Eosin (H&E) Staining

After mice were sacrificed, tumor tissues were collected. After that, they were fixed in 4% paraformaldehyde for 24 h, washed with PBS, and then embedded in paraffin. Then, 2-micrometer sections were stained with hematoxylin and eosin according to standard procedures.

### 2.14. Statistical Analysis

Data analysis was performed using SPSS v 24.0 (IBM Corp., Armonk, NY, USA) for Windows software (SPSS Inc., Chicago, IL, USA). Pearson’s χ^2^ test, Student’s *t*-test, and Fisher’s exact test were used to evaluate the relationship between SNHG4 expression and clinicopathological characteristics in endometrial cancer. To evaluate the performance in terms of the discriminative ability of the model, we used the x^2^ value of the log-rank test in the receiver operating characteristics (ROC) analysis. Progression-free survival (PFS) was analyzed using the Kaplan–Meier method. A log-rank test was used to estimate the pi between groups. The staged Cox proportional hazards model was used for multivariate survival analysis of significant variables in a single randomized analysis. Statistical tests were considered two-sided, and a *p*-value of 0.05 was considered statistically significant.

## 3. Results

### 3.1. Expression of SNHG4 in Endometrial Cancer Serum Correlates with Poor Prognosis

**Figure 1:**
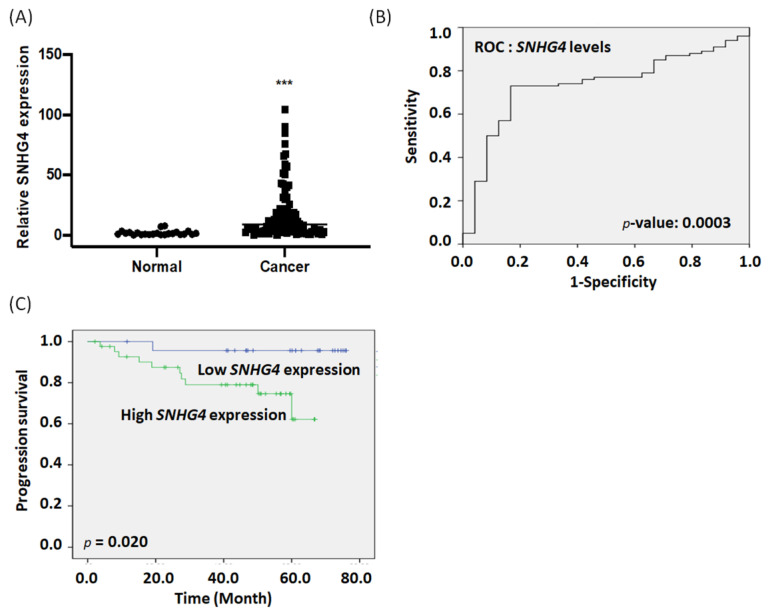
SNHG4 expression in human endometrial cancer patient serum. (**A**) Relative expression of SNHG4 was significantly higher in endometrial cancer (EC) patient serum (*n* = 100) than that in noncancerous patient serum (*n* = 26). (**B**) Receiver operating characteristic (ROC) curve for prognosis prediction of patients using SNHG4 level. The area under curve (AUC) is shown in plots. (**C**) Relative SNHG4 expression and its clinical significance based on Kaplan–Meier progression-free survival curves of patients with endometrial cancer and different levels of SNHG4. Data are expressed as mean ± standard deviation. *** *p* < 0.001 vs. non-tumor control.

We investigated whether the expression of SNHG4 in endometrial cancer serum (*n* = 100) and normal serum (*n* = 26) is related to the clinical and pathological characteristics of endometrial cancer. The expression of SNHG4 in endometrial cancer serum was more than 20 times higher than in noncancerous serum (*p* = 0.00001, Figure 1A). The area under the predicted curve (AUC) of the risk model for the SNHG4 data was 0.734 (*p* = 0.0003, Figure 1B). To further understand the clinical relevance of SNHG4 expressions in endometrial cancer serum, we divided all 100 patients with high SNHG4 expression (*n* = 73) and patients with low SNHG4 expression (*n* = 27) (Supplementary Table S3). Kaplan–Meier survival analysis showed that endometrial cancer patients with low SNHG4 expression had longer progression-free survival than did patients with high SNHG4 expression (*p* = 0.020, respectively, Figure 1C). Univariate and multivariate analysis using the Cox proportional hazards model confirmed that the expression of SNHG4 was a significant predictor of progression-free survival when univariate. (Univariate hazard ratio [HR] = 8.102 (1.014–64.758), *p* = 0.049) (Table 1). In addition, univariate proportional hazard analysis with lymph node metastasis (univariate hazard ratio [HR] = 8.138 (2.153–30.757), *p* = 0.002) showed that high expression of SNHG4 and lymph node metastasis were predictors.

### 3.2. SNHG4 Expression Is Increased in Endometrial Cancer Cell Lines and Correlates with Cell Proliferation, Invasion, Migration, and Colony Formation

**Figure 2:**
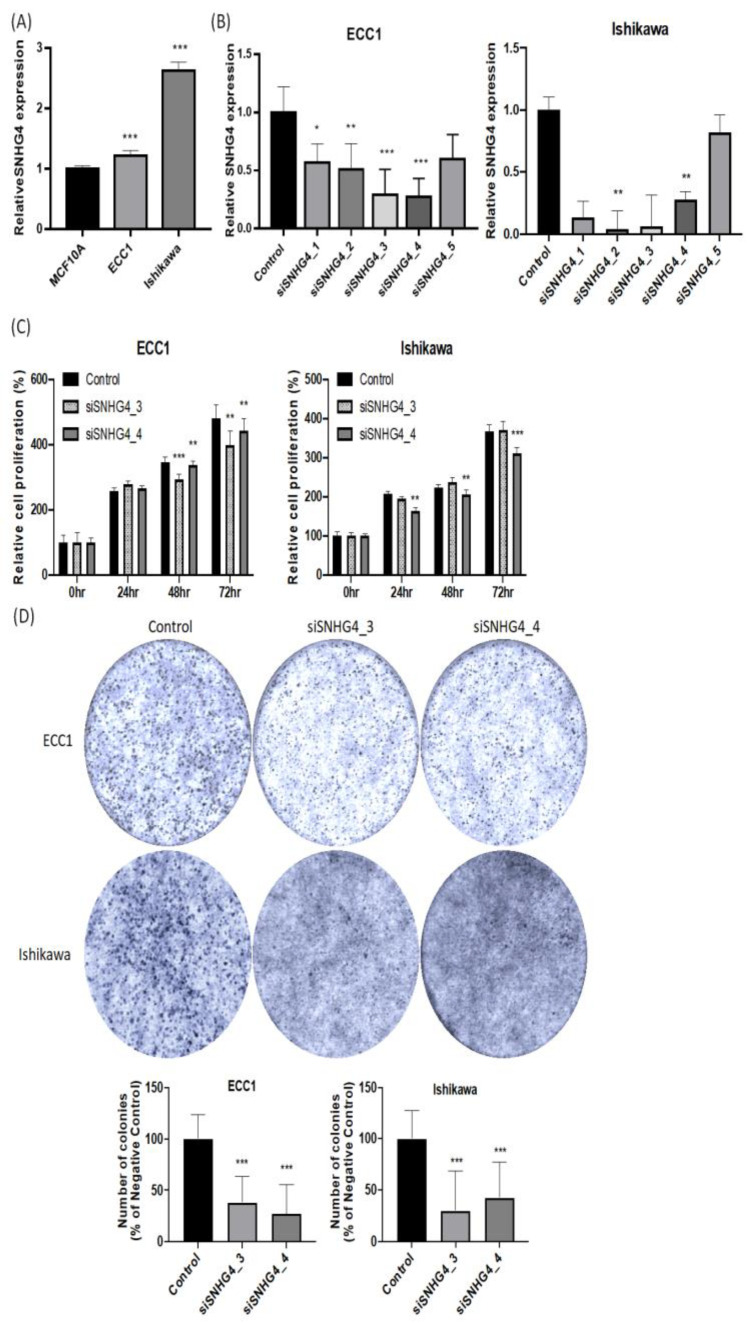

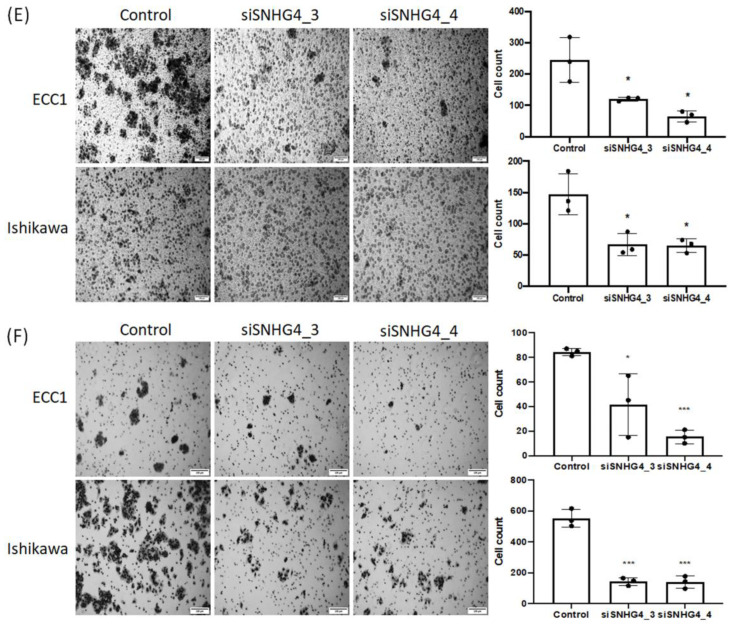
Knock-down of SNHG4 inhibits endometrial cancer cell proliferation, migration, invasion, and colonization. (**A**) Expression of SNHG4 in human epithelial cell (MCF10A) and human endometrial cancer cell lines as determined by quantitative real-time polymerase chain reaction (qRT-PCR). (**B**) Knock-down efficiency was determined by qRT-PCR analysis by transfecting cells with SNHG4 siRNA (siSNHG4: 30 nM) or control. (**C**) Knock-down of SNHG4 determined cell proliferation in ECC-1 and Ishikawa cells using the Cell Proliferation Cell Counting Kit-8. (**D**) Colony formation assays were performed in ECC1 and Ishikawa cells in which SNHG4 was knocked down. (**E**) Wound healing assay was used to determine migration in SNHG4-specific siRNA (siSNHG4)-transfected ECC-1 and Ishikawa cells. (**F**) Invasion was determined after 72 h in siSNHG4-transfected ECC-1 and Ishikawa cells using a Matrigel invasion assay. Scale bar, 100 μm. Bars represent mean ± standard deviation of three independent experiments performed in triplicate. * *p* < 0.05, ** *p* < 0.01 and *** *p* < 0.001 versus control.

To confirm the role of SNHG4 in endometrial cancer, we experimented with the expression of SNHG4 in the endometrial cancer cell lines. As shown in Figure 2A, it was confirmed that SNHG4 was expressed at a higher level in the two cell lines than in the control (MCF10A) cells. In order to explore the function of SNHG4 in the progression of endometrial cancer, siSNHG4 was transfected in both ECC1 and Ishikawa cells and knocked down, and the influence of the SNHG4 knock-down were inhibited assessed on the proliferation, migration, invasion, and metastasis of endometrial cancer cells. First, we designed five siRNAs targeting SNHG4 and then transfected them into ECC1 and Ishikawa cells. The subsequent qRT-PCR experiment demonstrated that all five siRNAs displayed various knockdown efficiencies (Figure 2B). Thus, we mainly used two siRNAs (Nos. 3 and 4) in the following studies. The two siSNHG4 cells in the endometrial cells showed inhibition of cell proliferation over time compared to the control (Figure 2C), and it was confirmed that siSNHG4 also decreased colony formation by more than 60% compared to the control (Figure 2D). Cell migration was assessed after 48 h using Transwell. It was confirmed that cell migration was reduced by more than 60% in siSNHG4 cells (Figure 2E). Cell invasion was assessed after 48 h using the Matrigel invasion assay. siSNHG4 cells were compared with control cells. siSNHG4 knock-down cells showed more than a 50% reduction in invasiveness (Figure 2F). Although uncontrolled cell proliferation is a biological feature characteristic of all tumors, a key pathophysiological feature, especially in malignancies, is the ability to progress through the natural tissue barrier. These results suggest that the expression of SNHG4 is associated with cancer growth and invasive metastasis.

### 3.3. SNHG4 Knock-Down Regulates the Cell Cycle in the G1 Phase

**Figure 3:**
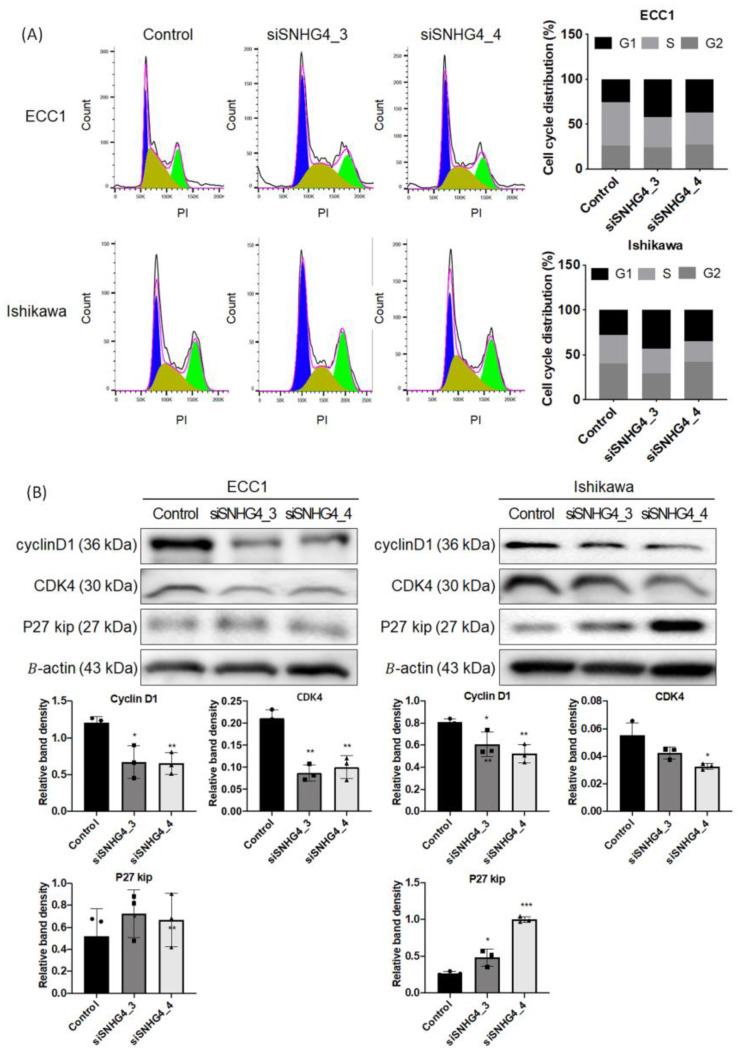
SNHG4 in endometrial cancer regulates the cell cycle. (**A**) Cell cycle analysis after SNHG4 knock-down for 48 h in endometrial cancer cells examined by PI staining assay by flow cytometry and results analyzed with Flow Jo 7.6.1 software. (**B**) Protein expression, including Cyclin D1, CDK4, and P27 kip in endometrial cancer cells, was detected by Western blot assay. Bars represent mean ± standard deviation of three independent experiments performed in triplicate. * *p* < 0.05, ** *p* < 0.01 and *** *p* < 0.001 versus control.

As cell proliferation is associated with the regulation of cell cycle progression, the cell cycle was assessed by flow cytometry in PI-stained cells to determine the effect of SNHG4 on cell cycle distribution. In ECC1 cells in which SNHG4 was knocked down for 48 h, it was confirmed that siSNHG4_3 increased by 40.3% and siSNHG4_4 by 32% compared to the control G1 phase (25.2%). In Ishikawa cells, siSNHG4_3 increased by 47.89% and siSNHG4_4 by 42.75% compared to the control G1 phase (29.3%) (Figure 3A). Western blot confirmed that knock-down of SNHG4 in two endometrial cancer cell lines regulates G1 arrest by CyclinD1, CDK4, and p27 (Figure 3B). In addition, to confirm cell cycle changes caused by SNHG4 knock-down, cell cycle markers were identified by Western blot at each timeline, and it was confirmed that CDK4 and CylinD1, which are G1 markers, were reduced by SNHG4 knock-down. It was confirmed that apoptosis was induced by G1 arrest, and it was confirmed that the expression of apoptosis markers increased in SNHG4 knock-down compared to control as time passed (Figure S1). As a result, we show indirectly that SNHG4 induces cell cycle arrest in G1 phase and induces apoptosis. 

### 3.4. SP-1 Expression Is Increased in Endometrial Cancer Cell Lines and Correlates with Cell Proliferation, Invasion, Migration, and Colony Formation

**Figure 4:**
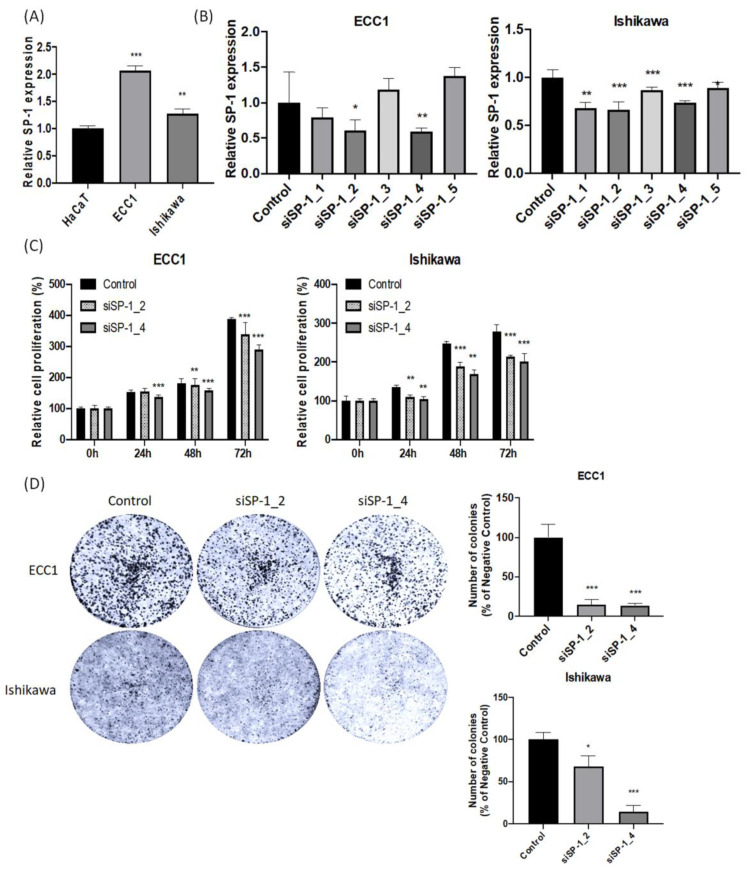

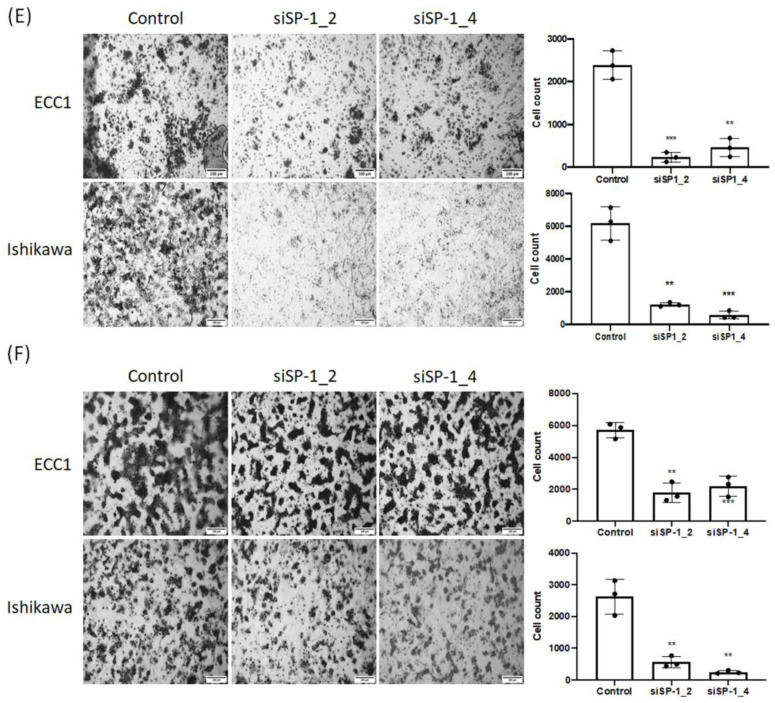
Knock-down of SP-1 inhibits endometrial cancer cell proliferation, migration, invasion, and colonization. (**A**) Expression of SP-1 in human keratinocytes (HaCaT) and human endometrial cancer cell lines as determined by quantitative real-time polymerase chain reaction (qRT-PCR). (**B**) Knock-down efficiency was determined by qRT-PCR analysis by transfecting cells with SP-1 siRNA (siSP-1: 30 nM) or control. (**C**) Knock-down of SP-1 determined cell proliferation in ECC-1 and Ishikawa cells using the Cell Proliferation Cell Counting Kit-8. (**D**) Colony formation assays were performed in ECC1 and Ishikawa cells in which SP-1 was knocked down. (**E**) Wound healing assay was used to determine migration in SP-1-specific siRNA (siSP-1) transfected ECC-1 and Ishikawa cells. (**F**) Invasion was determined after 72 h in siSP-1 transfected ECC-1 and Ishikawa cells using a Matrigel invasion assay. Scale bar, 100 μm. Bars represent mean ± standard deviation of three independent experiments performed in triplicate. * *p* < 0.05, ** *p* < 0.01 and *** *p* < 0.001 versus control.

Transcription factors (TFs) perform a key function in controlling lncRNA expression through sequence-specific binding sites on the lncRNA transcript. To find the transcription factor controlling the expression of SNHG4, SP-1 was found using the Alibaba 2.0 database, and the binding part was found using the JASPAR database (Figure S2). Next, expression of SP-1 was confirmed in the endometrial cancer cell lines ECC1 and Ishikawa. As shown in Figure 4A, it was confirmed that SP-1 was expressed at a higher level in the two cell lines than in the control (MCF10A) cells. In order to confirm the effect of SP-1 on endometrial cancer cells, siSP-1 was transfected in two cell lines and knocked down, and two siRNAs (Nos. 2 and 4) were selected (Figure 4B). In addition, qRT-PCR analyses revealed that siSP-1 depressed the endogenous SNHG4 expression (Figure S3A), which indicated that SP1 was required for the expression of SNHG4. The two siSP-1 cells in the endometrial cells showed inhibition of cell proliferation over time compared to the control (Figure 4C), and it was confirmed that siSP-1 also decreased colony formation by more than 60% compared to the control (Figure 4D). Cell migration was assessed after 48 h using Transwell. It was confirmed that cell migration was reduced by more than 70% in SP-1 knock-down cells (Figure 4E). Cell invasion was assessed after 48 h using the Matrigel invasion assay. SP-1 knock-down cells were compared with control cells. SP-1 knock-down cells showed more than a 70% reduction in invasiveness (Figure 4F). In summary, these data demonstrate that in endometrial cancer, SNHG4 is regulated by SP-1 at the transcriptional level and is indirectly associated with SNHG4.

### 3.5. SNHG4 Regulated EMT and SP-1 Expression in Endometrial Cancer Cells

**Figure 5:**
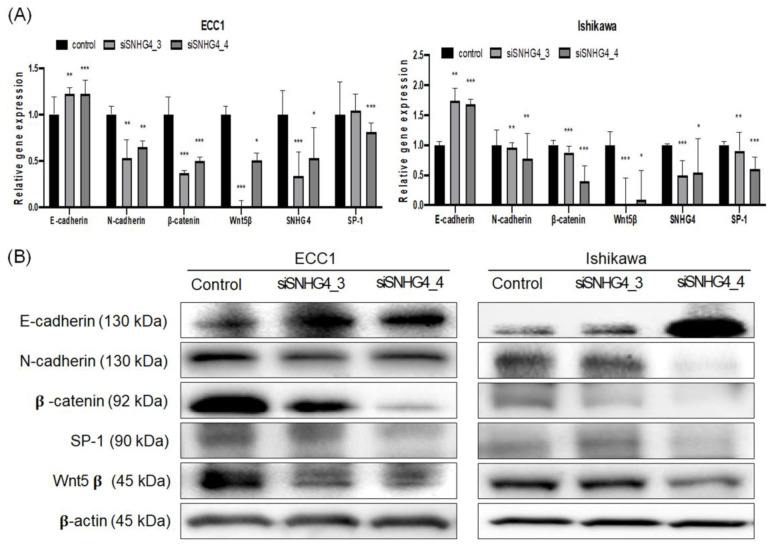
SNHG4 regulates EMT and SP-1 signaling pathways. (**A**) SNHG4 was knocked down in two endometrial cancer cell lines, and EMT and SP-1 expression were determined through quantitative real-time polymerase chain reaction (qRT-PCR) analysis. qRT-PCR was performed in triplicate. * *p* < 0.05, ** *p* < 0.01, *** *p* < 0.001 versus control. (**B**) Protein expression of protein lysates of two endometrial cancer cell lines in which SNHG4 was knocked down was confirmed by Western blot analysis.

EMT, which is important for cell migration and invasion, is a major factor in cancer, and identification of these factors may have clinical implications. Therefore, we identified a relationship between SNHG4 and EMT. mRNA levels were evaluated after knock-down of SNHG4 in two endometrial cancer cell lines, and it was confirmed that the expression of E-cadherin increased and, conversely, the expression of β-catenin, N-cadherin, SP-1, and Wnt5β decreased (Figure 5A). Protein expression was assessed using Western blot (Figure 5B). It was confirmed that the expression of E-cadherin increased in the two cells of endometrial cancer and, on the contrary, the protein expression of β-catenin, N-cadherin, SP-1, and Wnt5β decreased. Next, we confirmed the relationship between SP-1 and EMT because SP-1 also affected migration and invasion in endometrial cancer. After transfection with siSP-1 in both ECC-1 and Ishikawa cells, EMT-related markers were evaluated at the mRNA and protein levels. As a result, the expression of E-cadherin increased, whereas β-catenin, N-cadherin, and Wnt5β decreased (Figure S3B,C). These results indicate that dysregulation of EMT-related genes may be related to SNHG4-mediated effects on endometrial cancer cell migration and invasion. It also suggests that SP-1 and SNHG4 are involved in endometrial cancer cell migration and invasion.

### 3.6. SNHG4 Regulates Tumor Growth in a Xenograft Nude Mouse Model

**Figure 6:**
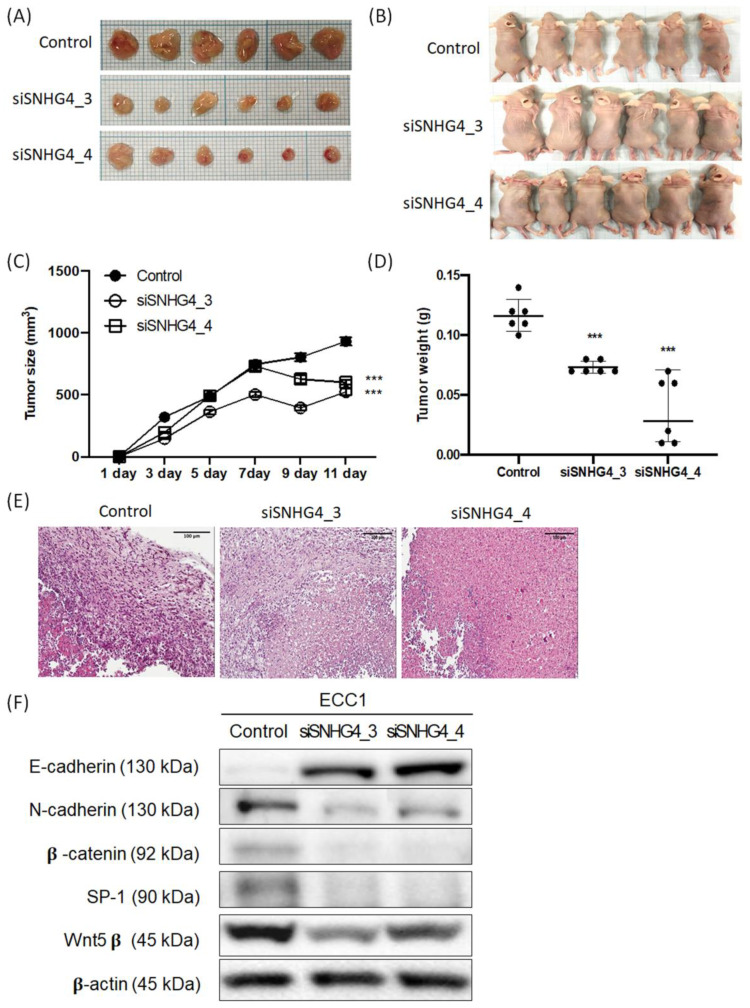
Effects of SNHG4 on tumor growth in vivo. ECC1 cells that knock down SNHG4 were subcutaneously injected into the right scapula region of nude mice. (**A**) Representative gross images of tumor masses from all mice in each group. (**B**) Total images of all mice in each group. (**C**) Tumor volumes were calculated every 2 days. Data are organized as mean ± SE (*n* = 6). *** *p* < 0.001 versus control. (**D**) Tumor weight graph. (**E**) Hematoxylin and eosin (H&E) staining of SNHG4 knock-down ECC1 cells (×200). (**F**) Expression of EMT and SP-1 signaling was measured by Western blotting in xenograft model tumor tissue.

To investigate whether SNHG4 could affect tumor growth in vivo, ECC1 cells with knock-down of SNHG4 were xenografted into a nude mouse model. Mice were sacrificed 11 days after transplantation of knock-down cells, and the overall tumor and appearance of the harvested mice are shown in Figure 6A,B. It was confirmed that the tumor volume and weight decreased in the SNHG4 knock-down group compared to the control group (Figure 6C,D). Histological examination by hematoxylin and eosin tissue staining revealed that the SNHG4 knock-down xenografts had smaller nucleoli and irregular nuclear membranes compared to the control group (Figure 6E). Tissues obtained from xenograft mice were utilized to evaluate the protein expression of EMT and SP-1 by Western blotting (Figure 6F). Compared to the control group, it was confirmed that the expression of E-cadherin increased in the SNHG4 knock-down group and, on the contrary, the expression of N-cadherin, β-catenin, SP-1, and Wnt5β decreased. These findings suggest that SNHG4 regulates tumor growth in vivo, further supporting the hypothesis that SNHG4 is involved in the malignant transformation of endometrial cancer cells.

## 4. Discussion

The lncRNA SNHG4, one of the members of the small nucleolar RNA host gene (SNHG) family, is known as a host gene for snoRNA present in the nucleus and cytoplasm [20]. So far, it has been reported that the SNHG family has 22 members, from SNHG1 to SNHG22 [10]. Among the 22 SNHG families, SNHG5, SNHG7, SNHG12, and SNHG16 are all known to promote cancer progression [21,22,23,24]. SNHG4 is mainly thought to play an oncogenic role in tumors, and although research in human diseases is increasing, it still does not cover all diseases. There is also a clear gap between existing studies and clinical data. In recent years, more and more studies are reporting that lncRNAs can be used as novel diagnostic and prognostic biomarkers.

Epithelial–mesenchymal metastasis in cancer cells plays an important role in invasion and metastasis and is also a promising therapeutic target in endometrial cancer [25]. However, the pre-selection of specific targets is challenging because of the complexity, diversity, and context of the signals affecting EMT regulation and the involvement of multiple factors [26]. Although more and more tumor-associated lncRNAs are being identified, the potential mechanisms involved in lncRNA dysregulation remain largely unclear. Studies have shown that transcription factors are involved in regulating some lncRNAs [27,28], and several transcription factors such as SP-1, ELK1, and E2F1 have been found to regulate lncRNAs in various tumors [29,30,31,32,33]. However, related studies on the association of transcription factors and lncRNA in endometrial cancer are limited.

In this study, we confirmed that the lncRNA SNHG4 is overexpressed in the blood of endometrial cancer patients using RT-PCR analysis. Then, we first showed that high expression of SNHG4 was strongly associated with tumor stage, lymph node metastasis, and short overall survival, suggesting that this lncRNA SNHG4 exerts a positive regulatory effect on the clinical progression of endometrial cancer. We further performed a loss-of-function assay to determine the potential function of SNHG4 and found that knockdown of SNHG4 markedly inhibited the proliferation, migration, and invasion of endometrial cancer cells, indicating that SNHG4 inhibits endometrial cancer cells. It suggests that it acts as a tumor promoter in progression. In addition, we evaluated whether the EMT signaling pathway is impaired upon SNHG4 knock-down. Loss of E-cadherin, an important event in EMT, and N-cadherin, which reduces the intercellular binding between two adjacent endothelial cells, reduces the intercellular binding between cells, thereby migrating cancer cells. In addition, β-catenin mobilizes and attenuates the relevant mesenchymal phenotype It was confirmed that the expression of E-cadherin increased in endometrial cancer cells in which SNHG4 was knocked down, and the expression of N-cadherin, β-catenin, and Wnt5β decreased. Additionally, to evaluate the relationship between SP-1 and SNHG4, one of the transcription factors regulating lncRNA [34], the expression of SP-1 was evaluated in endometrial cancer cells in which SNHG4 was knocked down, and it was confirmed that the expression of SP-1 was reduced. Thus, SNHG4 may contribute to the endometrial cancer cell phenotype through activation of EMT and SP-1 signaling.

Cell proliferation is one of the most basic properties of an organism. It is a process that maintains life and also plays an important role in the initiation and development of cancer [35,36]. Ki67, Cyclin D1, CDK1, CDK4, CDK6, and PCNA are all proliferation-associated proteins [37], and, among them, CyclinD1 is a G1 checkpoint protein [38]. CDK1, CDK4, and CDK6 are cyclin-dependent kinases that phosphorylate proteins to induce cell cycle processes and thus affect cell proliferation. SNHG4 knock-down is known to decrease cell viability and promote cell cycle arrest. In this study, it was confirmed that the expression of CyclinD1 and Cdk4 was decreased due to SNHG4 knock-down in endometrial cancer cells, and the arrest of the G1 phase was confirmed.

Using a xenograft mouse model, the functional role of SNHG4 has been studied in gastric, prostate, cervical cancer, and endometriosis [39]. Because of this, it is known that SNHG4 promotes the growth of several cancer cells and endometriosis cells in vivo. In this study, it was confirmed that cancer cell growth was inhibited by knock-down of SNHG4 in a xenograft mouse model of endometrial cancer cells. This suggests that SNHG4 is involved in the growth of endometrial cancer cells as well as endometriosis, a normal tissue. To date, there have been several studies on SNHG4 and cancer, but they are mainly in the basic research stage, and more clinical application studies are needed in the future. 

Recently, with the development of precision medicine in endometrial cancer, a new therapeutic approach based on molecular profiling is provided. In 2021, molecular classification was recommended by the National Comprehensive Cancer Network (NCCN) to improve the outcome of patients with endometrial cancer to select appropriate treatment regimens. Nevertheless, integrated classification is expensive, and the procedure is complicated, so its application is limited. In particular, the diagnosis of early stage endometrial cancer may be difficult due to its asymptomatic characteristics. As a biomarker for cancer diagnosis, treatment, and prognosis, lncRNAs can be an alternative for early detection, and profiling using lncRNAs can be a promising alternative. The reason is because the expression profile can offer more predictive information for cancer diagnosis than profiling hundreds and thousands of targeting mRNAs or proteins. 

In the present study, using clinical samples, it was confirmed that the expression of lncRNA SNHG4 was high in the blood of endometrial cancer patients. According to the cut-off value of SNHG4, we demonstrated that the high-risk set displayed a poor survival rate. In addition, we found that SNHG4 could be a potential therapeutic strategy given its mechanistic role in promoting tumor invasion, cell proliferation, and colony formation by regulating EMT and SP-1 signaling pathways.

## 5. Conclusions

Our study demonstrates that the lncRNA SNHG4 can act as a regulator of endometrial cancer cell progression. In this study, we investigated the anticancer properties of lncRNA SNHG4 in endometrial cancer cells and confirmed that SNHG4 knock-down inhibited EMT and SP-1, thereby inhibiting cell migration and invasion. In addition, it was clinically confirmed that SNHG4 is a biomarker that can predict stage and lymph node metastasis in endometrial cancer. Our study may provide a basis for endometrial cancer replacement therapy.

## Figures and Tables

**Table 1:** Univariate and multivariate analysis of various factors for progression-free survival.

	PFS			
	Univariate Analysis		Multivariate Analysis	
	HR (95% CI)	*p*-Value	HR (95% CI)	*p*-Value
SNHG4 expression	8.102 (1.014–64.758)	0.049	4.851 (0.538–43.757)	0.159
Age, years (continuous)	1.021 (0.956–1.091)	0.529	1.028 (0.932–1.134)	0.58
FIGO stage	2.462 (1.347–4.502)	0.003	2.079 (0.517–8.350)	0.302
Grade	1.374 (0.662–2.852)	0.394	1.019 (0.371–2.802)	0.97
Lymph node metastasis	8.138 (2.153–30.757)	0.002	3.068 (0.248–38.013)	0.383
Lymphovascular invasion	2.927 (0.855–10.024)	0.087	0.376 (0.069–2.046)	0.258
BMI	0.541 (0.158–1.853)	0.328	0.438 (0.095–2.022)	0.29
Tumor size	2.170 (0.920–5.120)	0.077	1.086 (0.362–3.257)	0.883

## Data Availability

Not applicable.

## Supplementary Materials

The following supporting information can be downloaded at: https://www.mdpi.com/article/10.3390/biomedicines11041018/s1, Table S1: Target siRNA sequences; Table S2: Real-time PCR target primer sequences; Table S3: Clinicopathologic factors in benign Endometrial serum and Endometrial cancer serum samples; Figure S1: Western blot analysis of cell cycle regulatory marker(A) and apoptosis marker(B) following siSNHG4 treatment of endometrial cancer cells. 1: control 2: siSNHG4_3 3: siSNHG4_4; Figure S2: SP-1 binding site using JASPAR database; Figure S3: SP-1 regulates EMT signaling pathways. (A) SP-1 was knocked down in two endometrial cancer cell lines. (B) EMT expression were determined through quantitative real-time polymerase chain reaction (qRT-PCR) analysis. qRT-PCR was performed in trip-licate. * *p* < 0.05, ** *p* < 0.01, *** *p* < 0.001 versus control. (C) EMT expression of protein lysates of two endometrial cancer cell lines in which SP-1 was knocked down was confirmed by Western blot analysis; Figure S4: The whole Western blot in Figure 3; Figure S5: The whole Western blot in Figure 5; Figure S6: The whole Western blot in Figure 6.
