# Supplementary material for: LncRNA SNHG4 Modulates EMT Signal and Antitumor Effects in Endometrial Cancer through Transcription Factor SP-1
Source: Biomedicines. 2023 Mar 27;11(4):1018. doi: 10.3390/biomedicines11041018 (PMC10135476; doi:10.3390/biomedicines11041018)
Supplement: Supplementary file 1 [file biomedicines-11-01018-s001.zip › biomedicines-2278460-supplementary.pdf]

Table S1. Target siRNA sequences

NO	siRNA	Sequence		Molecular weight
1	SNHG4_1	Sense	CAUCCUUCACCCAUCUGAAUU	6178
		Antisense	UUCAGAUGGGUGAAGGAUGUU	6458.2
2	SNHG4_2	Sense	CAUCUGAAGGGAGGAAAUAUU	6448.2
		Antisense	UAUUUCCUCCCUUCAGAUGUU	6173
3	SNHG4_3	Sense	GUCUUCUUGUCAUGUAGGUUU	6270
		Antisense	ACCUACAUGACAAGAAGACUU	6351.2
4	SNHG4_4	Sense	GUCAUGUAGGUCCCAAUUAUU	6299.1
		Antisense	UAAUUGGGACCUACAUGACUU	6322.1
5	SNHG4_5	Sense	CAAUCUCGGCUCACUACAAUU	6241.1
		Antisense	UUGUAGUGAGCCGAGAUUGUU	6395.2
6	SP-1_1	Sense	GCCUAAUAUUCAGUAUCAAUU	6267.1
		Antisense	UUGAUACUGAAUAUUAGGCUU	6324.1
7	SP-1_2	Sense	GGUCAAAUACAGAUCAUACUU	6329.2
		Antisense	GUAUGAUCUGUAUUUGACCUU	6277.1
8	SP-1_3	Sense	CAAUAGCUACUCAACUACUUU	6226.1
		Antisense	AGUAGUUGAGUAGCUAUUGUU	6380.2
9	SP-1_4	Sense	GCAACAUGGGAAUUAUGAAUU	6409.2
		Antisense	UUCAUAAUUCCCAUGUUGCUU	6197
10	SP-1_5	Sense	CCCUUAUUACCACCAAUAUUU	6163
		Antisense	AUAUUGGUGGUAAUAAGGGUU	6443.2
11	Control	Sense	CCUCGUGCCGUUCCAUCAGGUAGUU	7487.7
		Antisense	CUACCUGAUGGAACGGCACGAGGUU	7636.9

Table S2. Real-time PCR target primer sequences

		sequence 5' - 3'
E-cadherin	Forward	ATTCTGATTCTGCTGCTCTTG
	Reverse	AGTAGTCATAGTCCTGGTCCT
N-cadherin	Forward	CCCAAGACAAAGAGACCCAG
	Reverse	GCCACTGTGCTTACTGAATTG
β-catenin	Forward	TGCAGTTCGCCTTCACTATG
	Reverse	ACTAGTCGTGGAATGGCACC
Wnt5β	Forward	TGTGAGGTGAAGACCTGCTG
	Reverse	AAAGTTGGGGGAGTTCTCGT
SNHG4	Forward	GCAGGTGACAGTCTGCATGT
	Reverse	AAGAAGCTGTCATGGCCAAC
SP-1	Forward	CTGGTGGGCAGTATGTTGTG
	Reverse	TTGGTTTGCACCTGGTATGA

Table S3. Clinicopathologic factors in benign Endometrial serum and Endometrial cancer serum samples.

Factor	n (%)	Low	High	P-value[a]
Age (mean±SD)	100	51.14±8.86	53.64±10.45	0.553
Cell type				
Type I<br>Endometrioid adenocarcinoma	100	27	73	0.00001
Stage				0.855
I	64	18	46	
Ⅱ	9	2	7	
Ⅲ	22	5	16	
Ⅳ	5	2	3	
Grade				0.284
I	47	14	33	
Ⅱ	30	5	25	
Ⅲ	23	8	15	
Lymphnode metastasis				0.711
Yes	21	5	16	
No	79	22	57	
Lymphatic invasion				0.202
Yes	32	6	26	
No	68	21	47	
Tumor size				0.776
<2cm	32	10	22	
2_4	38	10	28	
≥4cm	30	7	23	
Recur				0.886
Yes	14	4	10	
No	86	23	63	
BMI				0.729
25>	49	25	35	
25<	51	13	38	

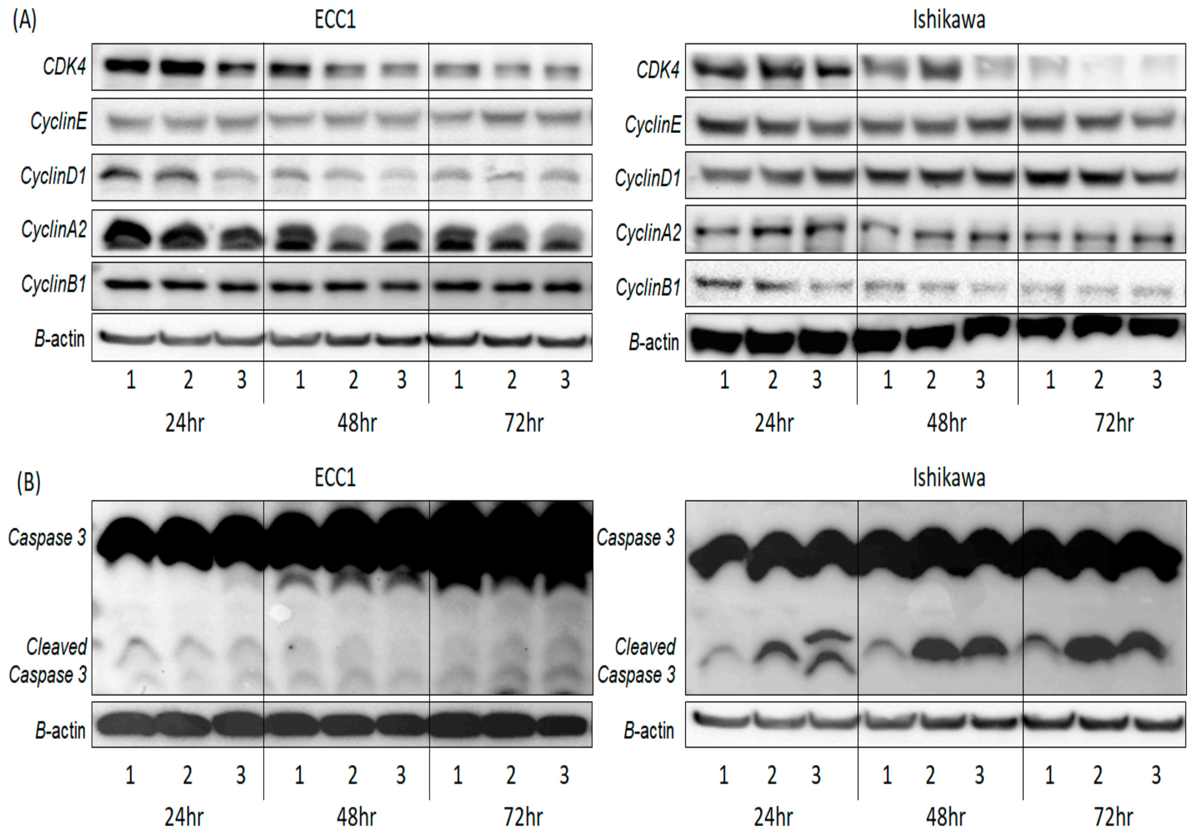

Figure S1. Western blot analysis of cell cycle regulatory marker(A) and apoptosis marker(B) following siSNHG4 treatment of endometrial cancer cells. 1: control 2: siSNHG4_3 3: siSNHG4_4

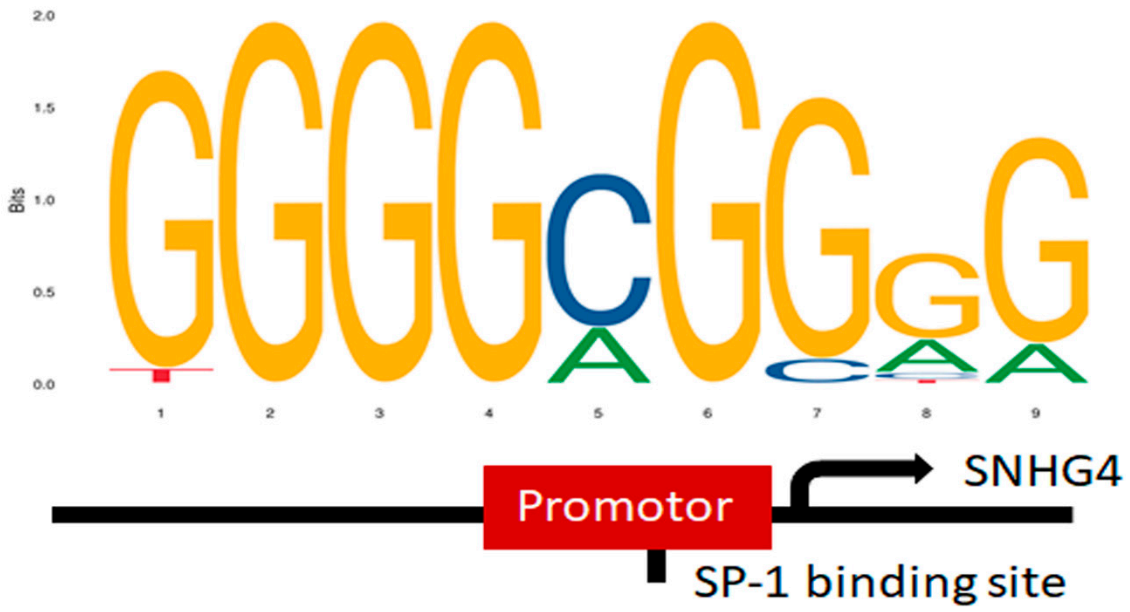

Figure S2. SP-1 binding site using JASPAR database.

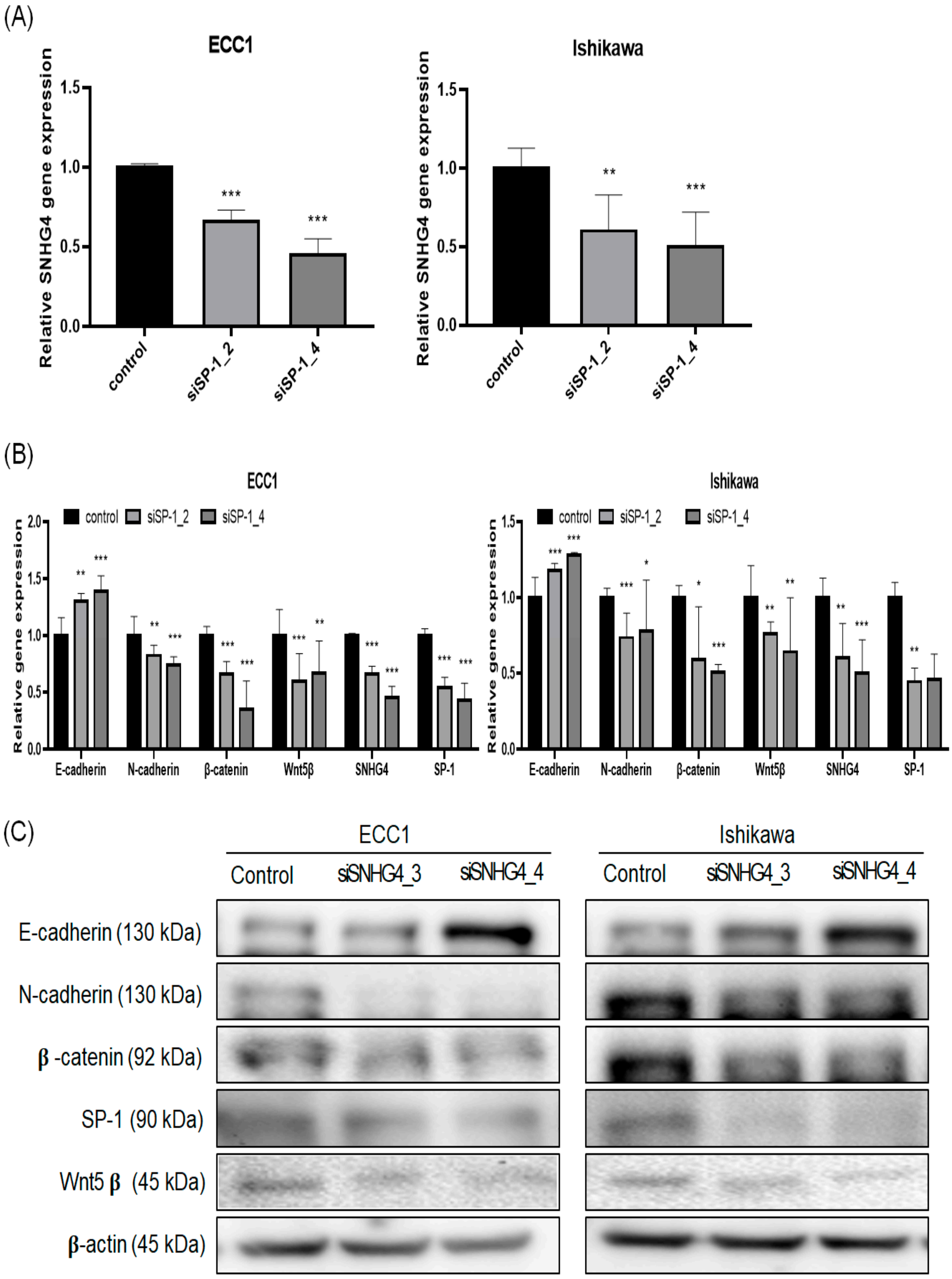

Figure S3. SP-1 regulates EMT signaling pathways. (A) SP-1 was knocked down in two endometrial cancer cell lines. (B) EMT expression were determined through quantitative real-time polymerase chain reaction (qRT-PCR) analysis. qRT-PCR was performed in trip-licate. * $p < 0.05$, *** $p < 0.01$, *** $p < 0.001$ versus control. (C) EMT expression of protein lysates of two endometrial cancer cell lines in which SP-1 was knocked down was confirmed by Western blot analysis.

Figure S4. The whole Western blot for Figure 3.

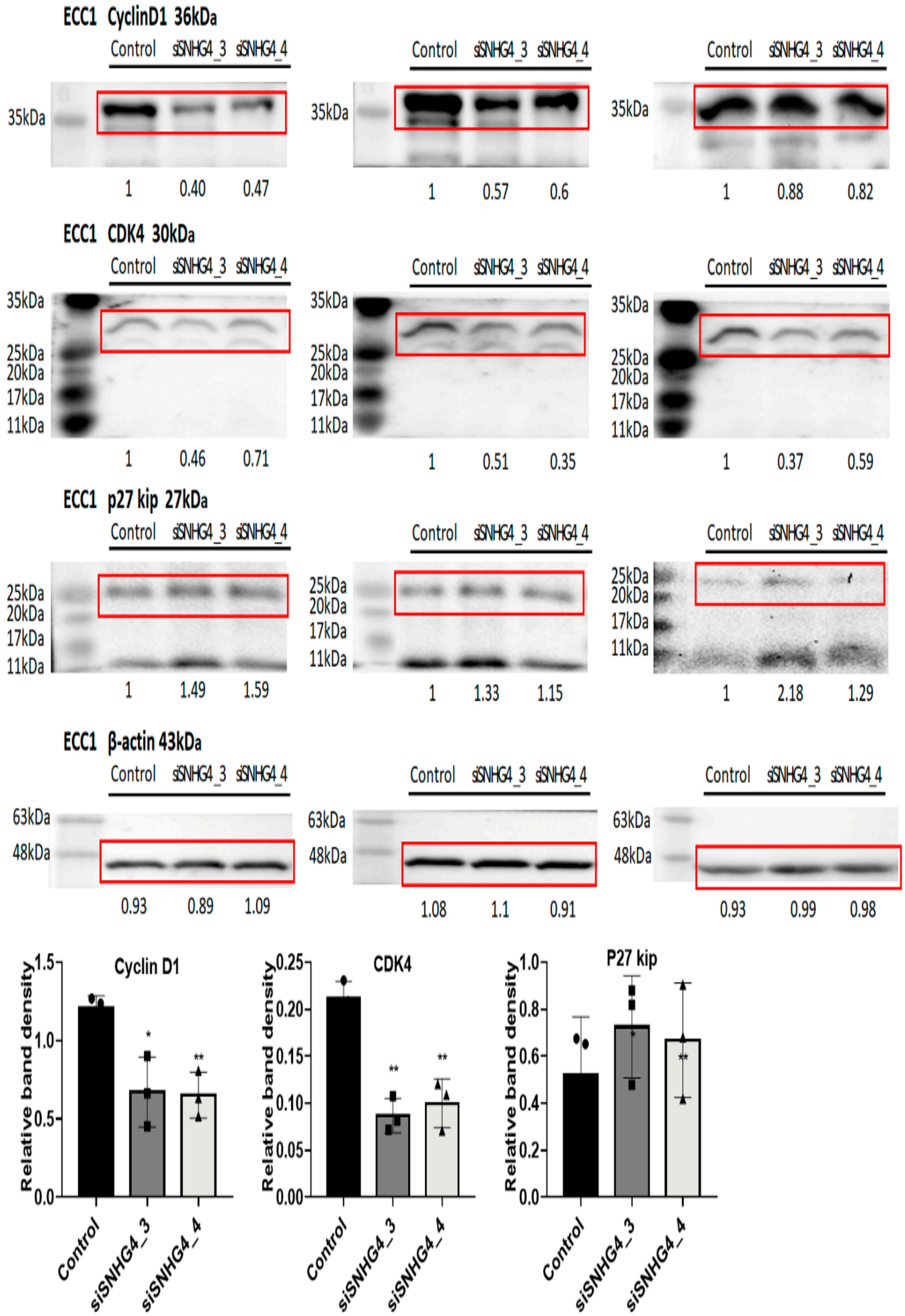

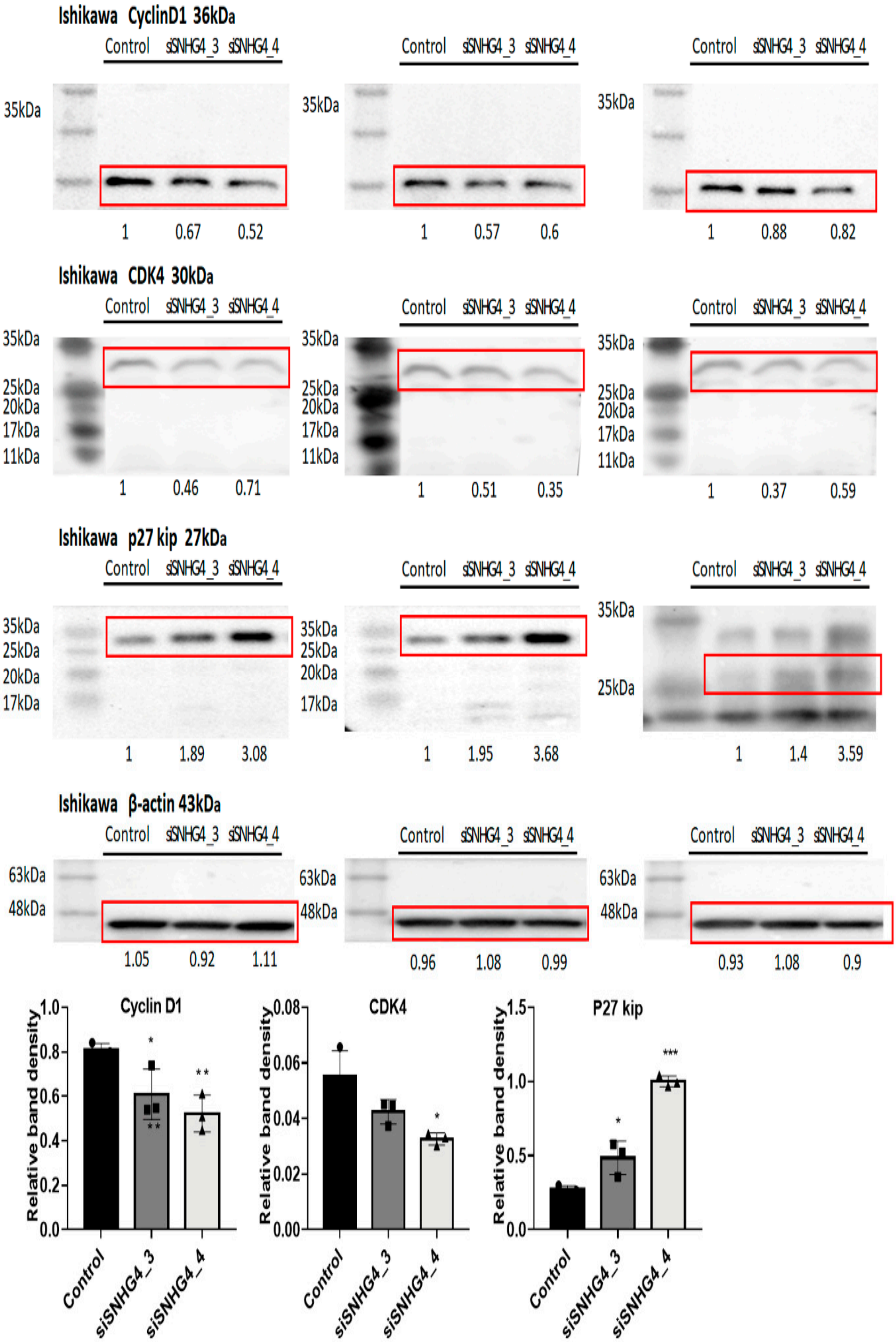
Ishikawa CyclinD1 36kDa
Control siSNHG4_3 siSNHG4_4
35kDa
1 0.67 0.52
1 0.57 0.6
1 0.88 0.82
Ishikawa CDK4 30kDa
35kDa 25kDa 20kDa 17kDa 11kDa
1 0.46 0.71
1 0.51 0.35
1 0.37 0.59
Ishikawa p27 kip 27kDa
35kDa 25kDa 20kDa 17kDa
1 1.89 3.08
1 1.95 3.68
1 1.4 3.59
Ishikawa β-actin 43kDa
63kDa 48kDa
1.05 0.92 1.11
0.96 1.08 0.99
0.93 1.08 0.9
Cyclin D1
CDK4
P27 kip
Relative band density
Control
siSNHG4_3
siSNHG4_4

Figure S5. The whole Western blot for Figure 5.

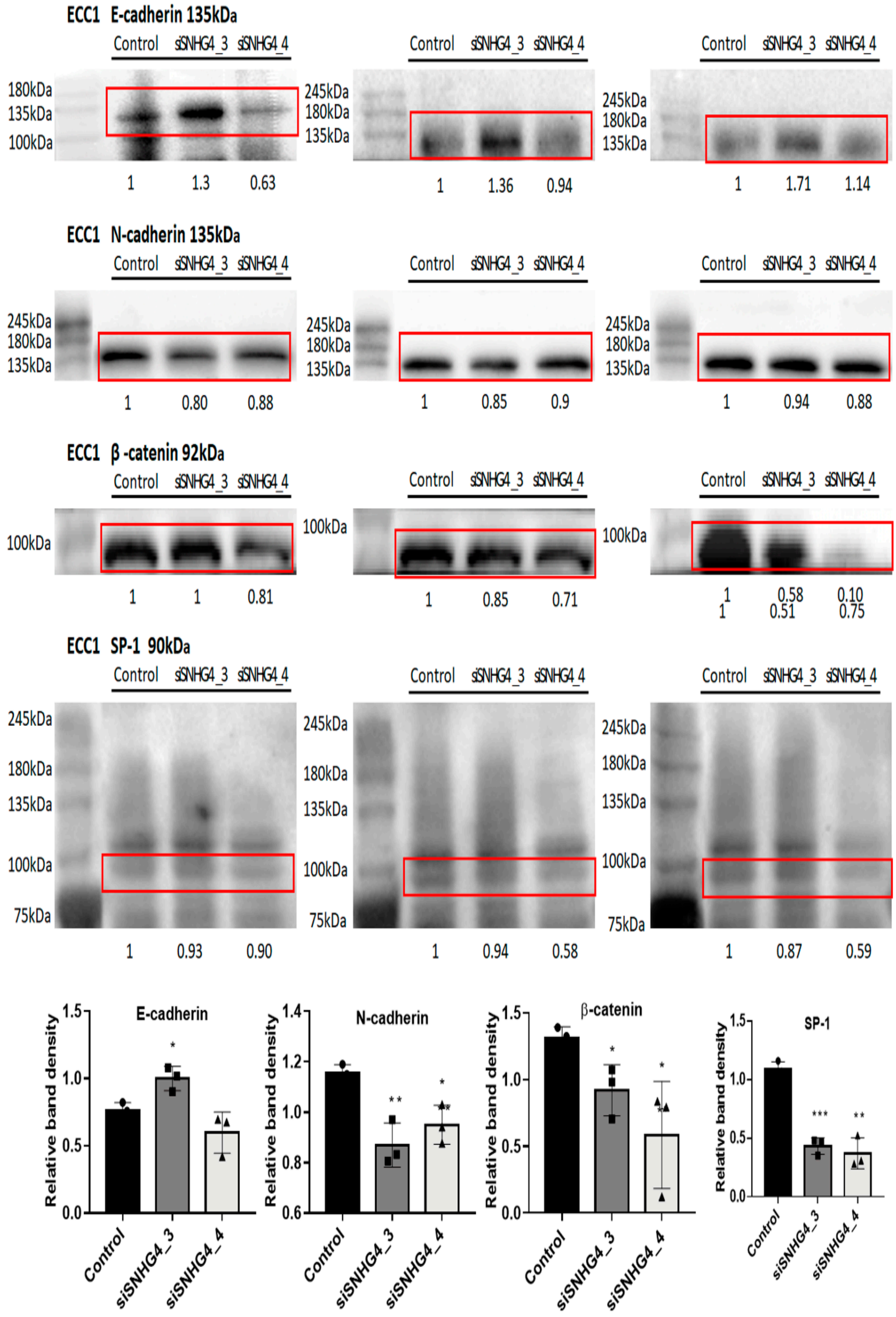

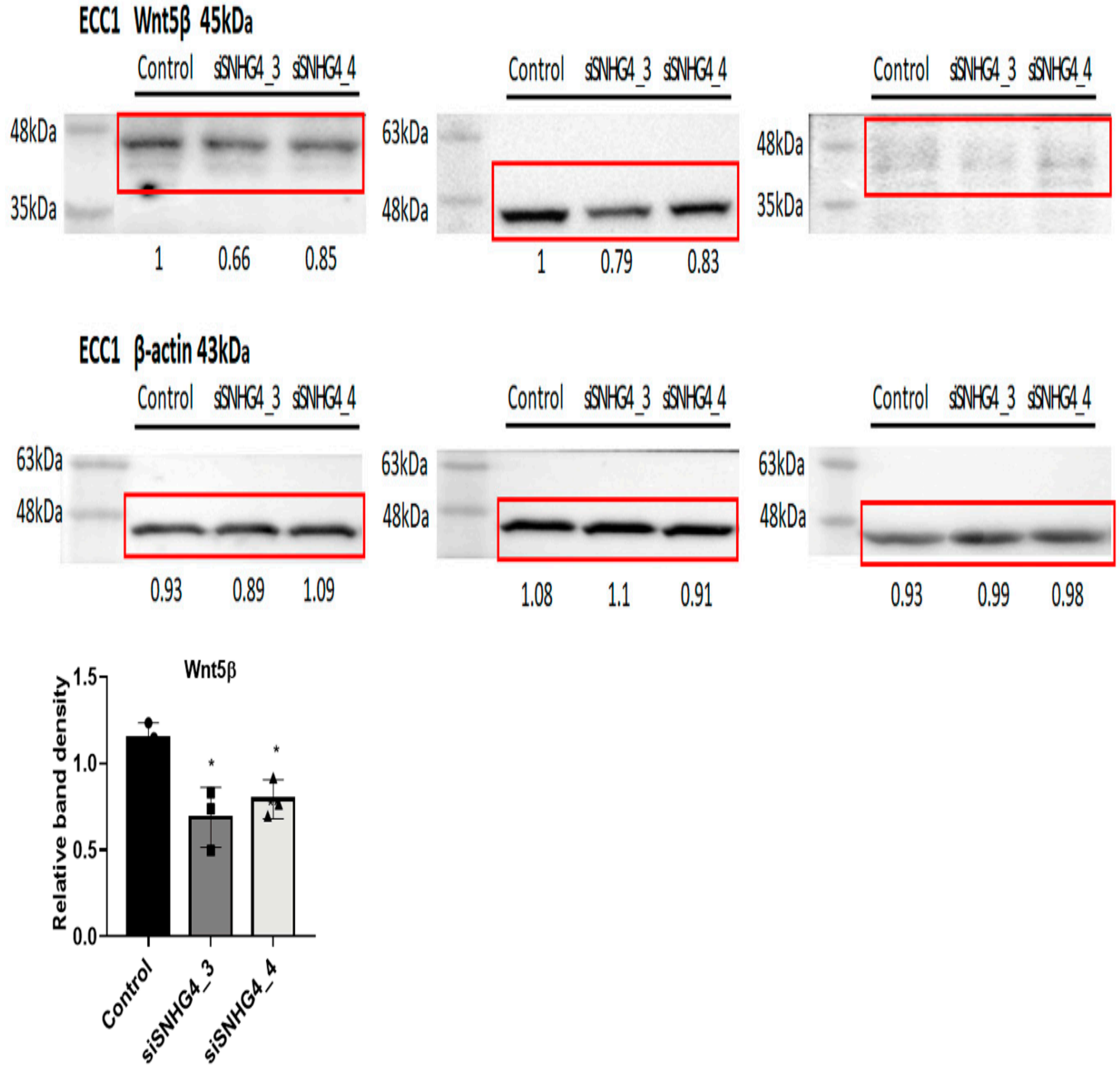

ECC1 Wnt5β 45kDa
Control siSNHG4_3 siSNHG4_4
48kDa
35kDa
63kDa
48kDa
1 0.66 0.85
1 0.79 0.83
ECC1 β-actin 43kDa
Control siSNHG4_3 siSNHG4_4
63kDa
48kDa
0.93 0.89 1.09
1.08 1.1 0.91
0.93 0.99 0.98
Wnt5β
Relative band density
1.5
1.0
0.5
0.0
*
*
Control
siSNHG4_3
siSNHG4_4

Figure S6. The whole Western blot for Figure 6.

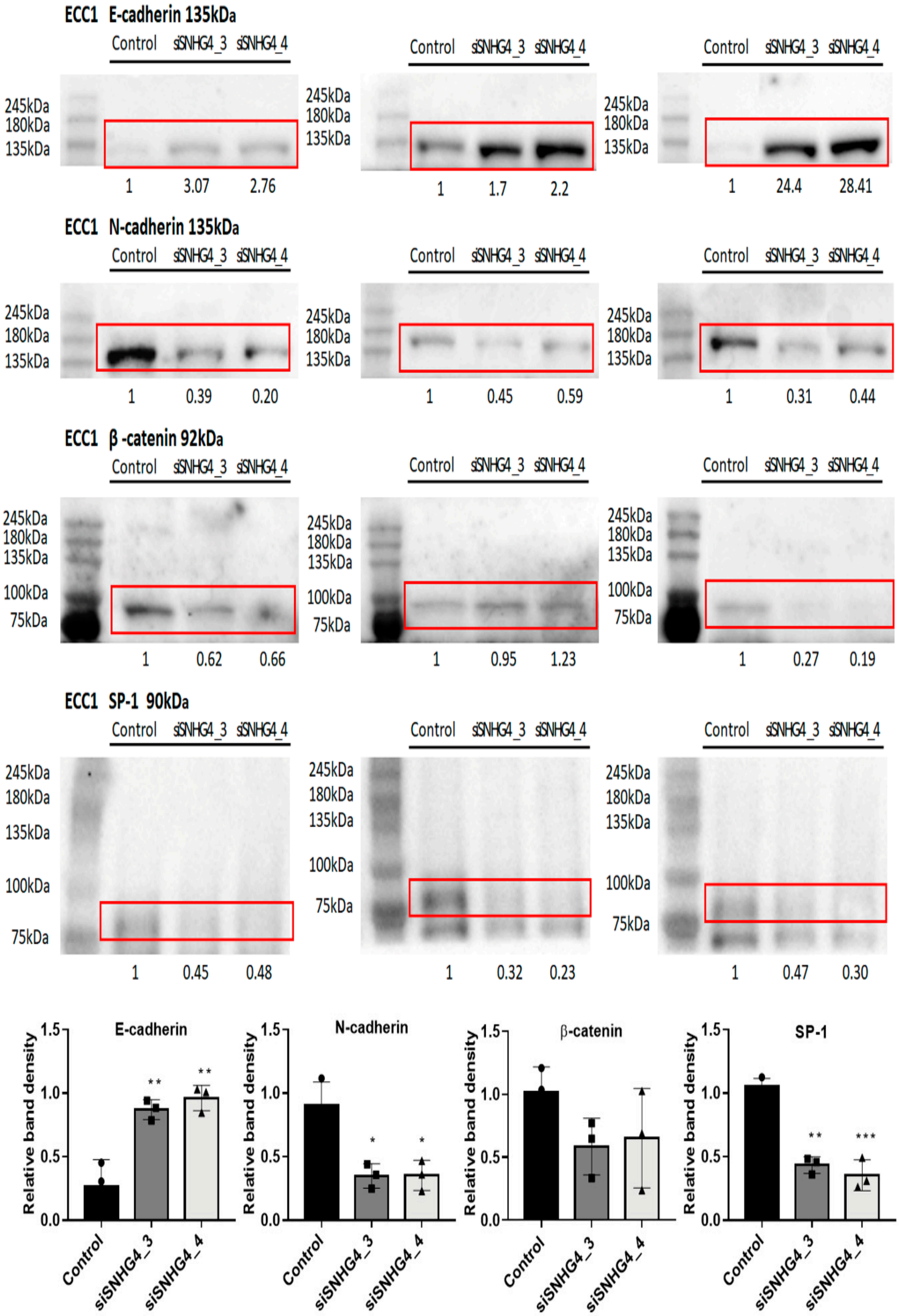

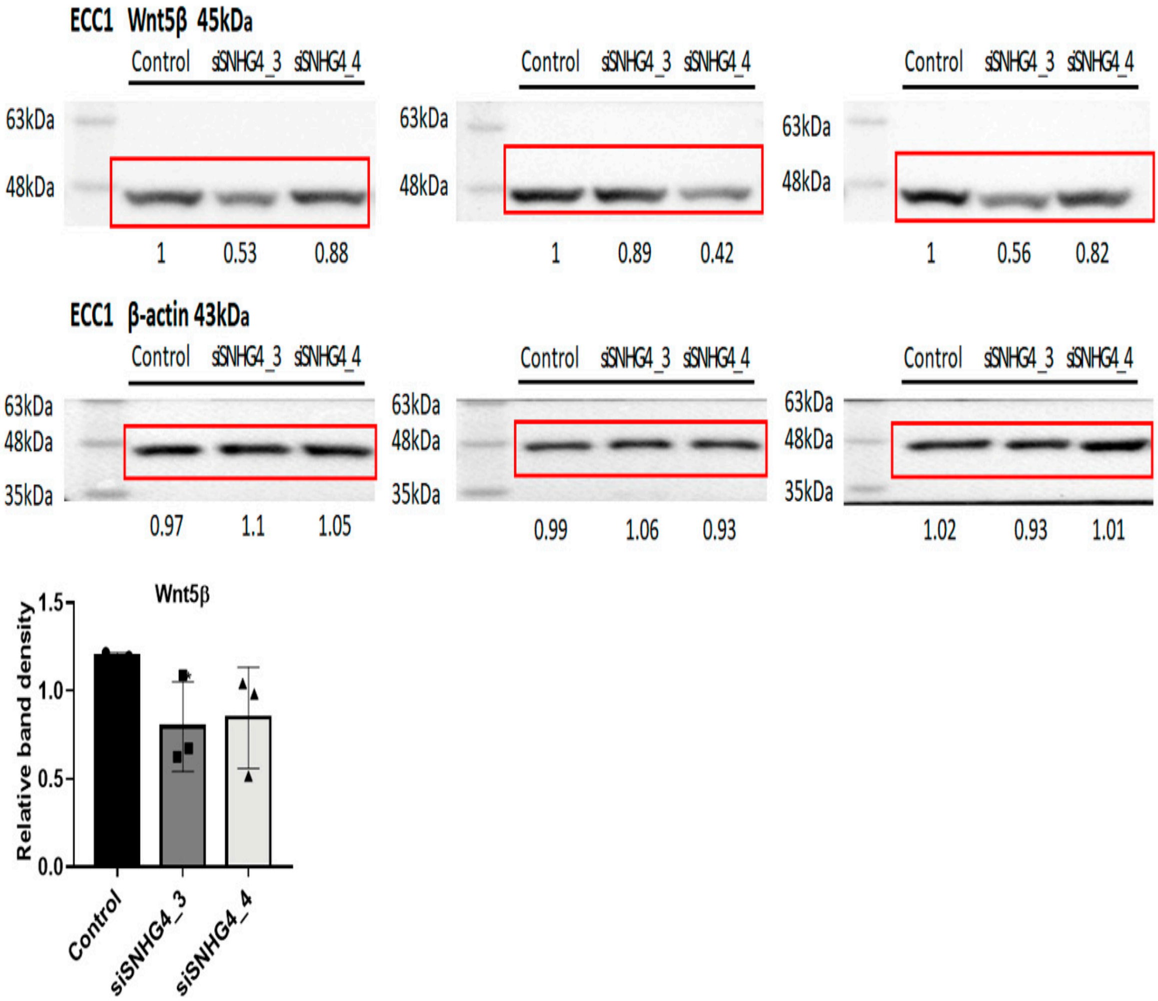

ECC1 Wnt5β 45kDa
Control siSNHG4_3 siSNHG4_4
63kDa
48kDa
1 0.53 0.88
1 0.89 0.42
1 0.56 0.82
ECC1 β-actin 43kDa
Control siSNHG4_3 siSNHG4_4
63kDa
48kDa
35kDa
0.97 1.1 1.05
0.99 1.06 0.93
1.02 0.93 1.01
Wnt5β
Relative band density
1.5
1.0
0.5
0.0
Control
siSNHG4_3
siSNHG4_4
